# Genomic comparisons of a bacterial lineage that inhabits both marine and terrestrial deep subsurface systems

**DOI:** 10.7717/peerj.3134

**Published:** 2017-04-06

**Authors:** Sean P. Jungbluth, Tijana Glavina del Rio, Susannah G. Tringe, Ramunas Stepanauskas, Michael S. Rappé

**Affiliations:** 1 Department of Oceanography, University of Hawaii at Manoa, Honolulu, HI, United States; 2 Center for Dark Energy Biosphere Investigations, University of Southern California, Los Angeles, CA, United States; 3 DOE Joint Genome Institute, Walnut Creek, CA, United States; 4 Single Cell Genomics Center, Bigelow Laboratory for Ocean Sciences, East Boothbay, ME, United States; 5 Hawaii Institute of Marine Biology, University of Hawaii at Manoa, Kaneohe, HI, United States

**Keywords:** Deep subsurface, Microorganisms, *Firmicutes*, Juan de Fuca Ridge, Chemoautotrophy, Basement biosphere, Sulfate reduction, Sporulation, Genomic, Metagenomic

## Abstract

It is generally accepted that diverse, poorly characterized microorganisms reside deep within Earth’s crust. One such lineage of deep subsurface-dwelling bacteria is an uncultivated member of the *Firmicutes* phylum that can dominate molecular surveys from both marine and continental rock fracture fluids, sometimes forming the sole member of a single-species microbiome. Here, we reconstructed a genome from basalt-hosted fluids of the deep subseafloor along the eastern Juan de Fuca Ridge flank and used a phylogenomic analysis to show that, despite vast differences in geographic origin and habitat, it forms a monophyletic clade with the terrestrial deep subsurface genome of “*Candidatus* Desulforudis audaxviator” MP104C. While a limited number of differences were observed between the marine genome of “*Candidatus* Desulfopertinax cowenii” modA32 and its terrestrial relative that may be of potential adaptive importance, here it is revealed that the two are remarkably similar thermophiles possessing the genetic capacity for motility, sporulation, hydrogenotrophy, chemoorganotrophy, dissimilatory sulfate reduction, and the ability to fix inorganic carbon via the Wood-Ljungdahl pathway for chemoautotrophic growth. Our results provide insights into the genetic repertoire within marine and terrestrial members of a bacterial lineage that is widespread in the global deep subsurface biosphere, and provides a natural means to investigate adaptations specific to these two environments.

## Introduction

Recent progress in understanding the nature of microbial life inhabiting the sediment-buried oceanic crust has been made through the use of ocean drilling program borehole observatories as platforms to successfully sample fluids that percolate through the subseafloor basement (Wheat et al., 2011). In 2003, a pioneering study by Cowen and colleagues (2003) used a passive-flow device to collect microbial biomass from fluids emanating out of an over-pressured borehole that originated from deep with the igneous basement of the eastern flank of the Juan de Fuca Ridge in the Northeast Pacific Ocean. Ribosomal RNA (16S rRNA) gene cloning and sequencing from the crustal fluids led to the first confirmation of microbial life in the deep marine igneous basement and revealed the presence of diverse bacteria and archaea. Discovered in this initial survey was an abundant, uniquely branching lineage within the bacterial phylum *Firmicutes* that was only distantly related to its closest known relative at the time, a thermophilic nitrate-reducing chemoautotroph isolated from a terrestrial volcanic hot spring, *Ammonifex degensii* (Huber et al., 1996).

Subsequent molecular surveys within both the terrestrial and marine deep subsurface revealed the presence of microorganisms related to the original marine firmicutes lineage (Lin et al., 2006; Jungbluth et al., 2013). In the deep subseafloor basement, this lineage has been recovered in high abundance (up to nearly 40%) from basaltic crustal fluids collected from a borehole nearby the initial location sampled ten years previously by Cowen and colleagues (2003), as well as from multiple boreholes spaced up to ∼70 km apart in the same region of the Northeast Pacific Ocean seafloor (Jungbluth et al., 2013; Jungbluth et al., 2014). In a surprising discovery, a single ecotype closely related to this firmicutes lineage was discovered in deep terrestrial subsurface fracture water of South Africa and found to be widespread (Magnabosco et al., 2014), where it sometimes made up an extremely high proportion of microorganisms *in situ* (Chivian et al., 2008). This lineage has since been found in other terrestrial habitats such as the Fennescandian Shield in Finland (Itävaara et al., 2011), a saline geothermal aquifer in Germany (Lerm et al., 2013), and an alkaline aquifer in Portugal (Tiago & Veríssimo, 2013). Based on 16S ribosomal RNA sequence analyses, most of the terrestrial and marine lineages form a monophyletic clade of predominantly subsurface origin but do not partition into subclades of exclusively terrestrial and marine origin, suggesting that there may have been multiple transitions between the terrestrial and marine deep subsurface environments (Jungbluth et al., 2013).

Chivian and colleagues (2008) reconstructed the first complete genome from a terrestrial member of this firmicutes lineage, provisionally named “*Candidatus* Desulforudis audaxviator” MP104C, via metagenome sequencing of a very low diversity sample from a deep gold mine in South Africa. The “*Ca.* D. audaxviator” genome revealed a motile, sporulating, thermophilic chemolithoautroptroph genetically capable of dissimilatory sulfate reduction, hydrogenotrophy, nitrogen fixation, and carbon fixation via the reductive acetyl-coenzyme A (Wood-Ljungdahl) pathway (Chivian et al., 2008). Thus, “*Ca.* D. audaxviator” appears well suited for an independent lifestyle within the deep continental subsurface environment. “*Ca.* D. audaxviator” and close relatives have continued to be recovered in subsequent metagenomes sequenced from the South African subsurface (Lau et al., 2014; Magnabosco et al., 2016). Recently, five flow-sorted and single amplified genomes related to “*Ca.* D. audaxviator” were sequenced from the terrestrial subsurface of South Africa, revealing significant genotypic variation with the terrestrial genomes and providing evidence for horizontal gene transfer and viral infection in the terrestrial subsurface environment (Labonté et al., 2015). To date, knowledge regarding marine members of this deep subsurface firmicutes lineage has been limited to phylogenetic (16S rRNA) and functional (dsr) gene surveys (Jungbluth et al., 2013; Robador et al., 2015).

In this study, we sought to improve understanding of the functional and evolutionary attributes of microorganisms inhabiting the deep subseafloor basement by sequencing the environmental DNA from two basement fluid samples from Juan de Fuca Ridge flank boreholes U1362A and U1362B, generating the first metagenomes from this environment. Binning of the resulting sequence data led to the reconstruction of a nearly complete genome closely related to “*Ca.* D. audaxviator.” This genome has allowed us to compare the functional composition of members of a microbial lineage that spans the terrestrial and marine deep subsurface, investigate its evolutionary history, and determine its prevalence within a globally-distributed assemblage of metagenomes.

## Materials and Methods

### Borehole fluid sampling

The methods used to collect samples during R/V Atlantis cruise ATL18_07 (28 June 2011–14 July 2011) are described elsewhere (Jungbluth et al., 2016). Briefly, basement crustal fluids were collected from CORK observatories located in 3.5 million-year-old ocean crust east of the Juan de Fuca spreading center in the Northeast Pacific Ocean. Basement fluids were collected from the polytetrafluoroethylene (PTFE) lined fluid delivery lines associated with the lateral CORKs (L-CORKs) at boreholes U1362A (47°45.6628′N, 127°45.6720′W) and U1362B (47°45.4997′N, 127°45.7312′W). These lines extend to 200 m and 30 m below the sediment-basement interface, respectively. Fluids were filtered *in situ* via a mobile pumping system (Cowen et al., 2012) through Steripak-GP20 filter cartridges (Millipore, Billerica, MA, USA) containing 0.22 µm pore-sized polyethersulfone membranes. A filtration rate of 1 L min^−1^ was calculated from laboratory tests, indicating that ∼124 L (U1362A) and ∼70 L (U1362B) of deep subsurface crustal fluids were filtered. Based on average cell abundances in whole water samples collected on the same dive/sampling sequence (Jungbluth et al., 2016), ∼2.6 × 10^9^ and ∼0.18 × 10^9^ cells were collected from U1362A and U1362B, respectively.

### DNA extraction and metagenome sequencing

Nucleic acids were extracted from borehole fluids using a modified phenol/chloroform lysis and purification method, and is described in detail elsewhere (Jungbluth et al., 2016; samples SSF21-22, SSF23-24). Library preparation, DNA sequencing, read quality-control, metagenome assembly, and gene prediction and annotation were conducted by the Department of Energy Joint Genome Institute as part of their Community Science Program using previously described informatics workflows (Huntemann et al., 2016), which are described in detail elsewhere (Jungbluth, Amend & Rappé, 2017).

### Genomic bin identification and reconstruction

All metagenomic scaffolds greater than 200 basepairs (bp) from U1362A (*n* = 137,672 contigs) and U1362B (*n* = 212,542 contigs) were binned separately with MaxBin v1.4 (Wu et al., 2014) using the 40 marker gene set universal among bacteria and archaea (Wu, Jospin & Eisen, 2013), minimum contig length of 1,000 bp, and default parameters. Contig coverage from each metagenome was estimated using the quality control-filtered raw reads as input for mapping using Bowtie2 v2.1.0 (Langmead & Salzberg, 2012) via MaxBin. The genomic bins were screened and analyzed for completeness, contamination, and assigned taxonomic identifications using CheckM v1.0.5 (Parks et al., 2015) with default parameters.

Raw quality control-filtered sequence reads from the U1362A and U1362B metagenomes related to “*Ca.* D. audaxviator” were identified by mapping to three sources: (1) a single genomic bin from U1362A related to “*Ca.* D. audaxviator” identified via CheckM (bin A32), (2) the “*Ca.* D. audaxviator” genome, (3) and all “*Ca.* D. audaxviator”-related contigs >200 bp from the U1362A and U1362B metagenome assemblies generated by the Joint Genome Institute. Mapping was performed independently for the U1362A and U1362B metagenomes using both the bbmap v34.25 (http://sourceforge.net/projects/bbmap/) and Bowtie2 v2.1.0 (Langmead & Salzberg, 2012) software packages with default parameters and the paired-end read-mapping feature (Table S1). All reads from the U1362A metagenome mapping to any of the three sources (1,785,284 sequences) were assembled using SPAdes v3.5.0 (Bankevich et al., 2012) with options –k: 21,33,55,77, --careful –pe1-12 and default parameters. Contaminating contigs in the assembly were screened and removed using the JGI ProDeGe web portal v2.0 (https://prodege.jgi-psf.org/) on April 10, 2015, using default parameters with the following taxonomy specified: “Bacteria; Firmicutes; Clostridia” (Tennessen et al., 2016). Contigs remaining following the use of ProDeGe comprise the genome bin henceforth named “*Ca.* Desulfopertinax cowenii” modA32 and were screened using CheckM as described above.

### Genome annotation and analysis

The modified genome bin resulting from the pipeline described above (“Ca. D. cowenii” modA32) was annotated via the Joint Genome Institute’s Integrated Microbial Genomes-Expert Review (IMG-ER) web portal (Markowitz et al., 2014; Huntemann et al., 2015). Annotations in the IMG-ER web portal served as the source of reported genome characteristics and reported genes and their assignment to COGs. Phylogenetically informative marker genes from “Ca. D. cowenii” were identified and extracted using the ‘tree’ command in CheckM. In CheckM, open reading frames were called using prodigal v2.6.1 (Hyatt et al., 2012) and a set of 43 lineage-specific marker genes, similar to the universal set used by PhyloSift (Darling et al., 2014), were identified and aligned using HMMER v3.1b1 (Eddy, 2011). Initial phylogenetic analysis used pplacer (v1.1.alpha16-1-gf748c91) (Matsen, Kodner & Armbrust, 2010) to place sequences into a CheckM tree/database (version 0.9.7) composed of 2,052 finished and 3,604 draft genomes (Markowitz et al., 2012).

An alignment 6,988 amino acids in length corresponding to the 43 concatenated marker genes from “*Ca.* D. cowenii,” “*Ca.* D. audaxviator,” other *Firmicutes,* and *Actinobacteria* were used for additional phylogenetic analysis. The concatenated amino acid alignment was used to generate a phylogeny using FastTree v2.1.9 (Price, Dehal & Arkin, 2010) with the WAG amino acid substitution model. The dendogram was visualized using iTOL v3 (Letunic & Bork, 2016).

Average nucleotide identity (ANI) was computed in IMG-ER using pairwise bidirectional best nSimScan hits of genes having 70% or more identity and at least 70% coverage of the shorter gene. The “*Ca.* D. cowenii” → [other genome] values are reported. Protein-coding genes in “*Ca.* D. cowenii” with homologs in “*Ca.* D. audaxviator,” and vice versa, were identified and percent similarity estimated using the “Phylogenetic Profiler” tool in IMG-ER with default parameters (max e-value: 10e^−5^; minimum identity: 30%). Average amino acid identity (AAI) was computed for pairs of genomes closely related to “*Ca.* D. cowenii” with an online web tool (http://enve-omics.ce.gatech.edu/aai/) using default parameters. All non-RNA genes at least 100 amino acids in length were used in this analysis. Two-way average amino acid identity scores are reported and the percent shared genes were calculated as follows: 100 × (2 × (number of proteins used for two-way AAI analysis))/((total number of amino acids ≥ 100 from genome A) + (total number of amino acids ≥ 100 from genome B)). Estimates of transposase and integrase abundance were derived in IMG using a functional profile of 100 pfams and COG functions selected searching for keywords “transposase” and “integrase.”

### Genome and scaffold visualizations

Global genome comparisons were visualized in Circos v0.67-5 (Krzywinski et al., 2009). Links between genomic regions of “*Ca.* D. cowenii” and “*Ca.* D. audaxviator” represent best reciprocal BLAST hits, which were generated using the blast_rbh.py script (https://github.com/peterjc/galaxy_blast/tree/master/tools/blast_rbh) with blastn v2.2.29 (Altschul et al., 1990) and default parameters. Links between genomic regions from the single amplified genomes of Labonté et al. (2015) represent BLAST hits that were generated using blastn with default parameters and using “*Ca.* D. cowenii” and “*Ca.* D. audaxviator” as reference databases.

Selected scaffold regions were visualized with Easyfig v2.2.2 (Sullivan, Petty & Beatson, 2011). Similarity between regions was assessed using BLAST wrapped within Easyfig using default parameters and task: blastn; minimum hit length: 50; max e-value: 0.001; minimum identity value: 50. In all instances of blast, contigs from “*Ca.* D. cowenii” were used as the query and “*Ca.* D. audaxviator” was used as the reference, with the exception of the single three-scaffold comparison where “*Ca.* D. audaxviator” was used as the query and “*Ca.* D. cowenii” Ga007115_16 used as the reference.

### Metagenome fragment recruitment

Quality-filtered raw reads from the U1362A metagenome were mapped to the six scaffolds that make up the “*Ca.* D. cowenii” genome bin and the “*Ca.* D. audaxviator” genome. Recruitment was performed using FR-HIT v0.7.1 (Niu et al., 2011) with default parameters (minimum sequence similarity 75%) and reporting a single best top hit for each read (-r 1).

### Analysis of metagenome-derived SSU rRNA genes

Full length SSU rRNA genes from the raw quality-filtered U1362A and U1362B metagenome reads were assembled using EMIRGE (Miller et al., 2011) with default parameters and -a 20, -i 270, -s 100, -l 150, -j 1.0, –phred33, and using the SILVA SSURef_Nr99 version 119 database that was prepared using the fix_nonstandard_chars.py script supplied on the EMIRGE website (https://github.com/csmiller/EMIRGE). Out of 1951 (U1362A) and 1434 (U1362B) near full-length SSU rRNA sequences constructed after 66 (U1362A) and 100 (U1362B) iterations of EMIRGE, a single sequence from U1362A related to the “*Ca.* D. audaxviator” lineage was identified through the SILVA online portal (Pruesse, Peplies & Glöckner, 2012). The sequence was aligned using the SINA online aligner and manually curated in ARB (Ludwig et al., 2004). Ambiguous and mis-aligned positions were excluded from further analysis.

A base SSU rRNA gene phylogenetic tree was reconstructed in ARB from 36 sequences and an alignment of 797 nucleotide positions using RAxML v7.72 (Stamatakis, 2006) with default parameters, the GTR+G+I nucleotide substitution model identified via JModelTest v2.1.1 (Darriba et al., 2012), and selecting the best tree from 100 iterations. Bootstrapping was performed in ARB using the RAxML tool with 2,000 replicates (Stamatakis, Hoover & Rougemont, 2008). Sequences of short length, including a masked version of the “*Ca.* D. audaxviator”-related SSU rRNA gene found here, were added to the phylogeny using the parsimony insertion tool in ARB and a filter containing 363 nucleotide positions.

### Phylogenetic analysis of *dsrAB* gene sequences

DNA sequences corresponding to dissimilatory sulfite reductase subunits alpha and beta (*dsrAB*) were aligned in ARB using the ‘integrated aligners’ tool and a previously published database of aligned *dsrAB* sequences (Loy et al., 2009). Additional sequences were identified and included via BLAST search of the non-redundant NCBI database using megablast and blastn with default parameters. Phylogenetic analyses were performed individually for *dsrA* and *dsrB* using RAxML with the GTR model of nucleotide substitution under the gamma- and invariable-models of rate heterogeneity, identified via jModelTest. The tree with the highest negative log-likelihood score was selected from performing 100 iterations using RAxML with default parameters. Phylogenies for the base trees were derived from partial length *dsrA* and *dsrB* alignments (545 and 303 nucleotides, respectively) and bootstrapping was performed in ARB using the RAxML rapid bootstrap analysis algorithm with 2,000 bootstraps.

### Analysis of global distribution patterns

All protein-coding genes corresponding to the genomes of “*Ca.* D. cowenii” (1,782 genes) and “*Ca.* D. audaxviator” (2,239 genes) were used to generate a profile against 489 globally-distributed metagenomes from marine subsurface fluids, the terrestrial subsurface, terrestrial hot springs, marine sediments, and seawater (Table S2). In IMG-ER, the “Profile & Alignment” tool was used to query assembled metagenomes using genes corresponding to the two genomes, a maximum e-value of 10^−5^, and a minimum similarity of 70%. The number of gene hits was converted to a relative frequency and the location of hits was visualized in R v3.1.2 (R Core Team, 2015) using latitude and longitude information provided as metadata and the R maps package (version 2.3-10).

Fragment recruitment was subsequently used in effort to discriminate between the distribution of the marine (“*Ca.* D. cowenii’ modA32A) and terrestrial (*“Ca.* D. audaxviator”) genomes of this *Firmicutes* lineage. Raw reads corresponding to IMG-ER metagenomes with the highest hit frequencies in the profiles generated in IMG, and additional unamplified metagenomes from the marine and terrestrial subsurface available only via NCBI sequence read archive and MG-RAST, were used as references for mapping to the genomes of “*Ca.* D. cowenii” and “*Ca.* D. audaxviator” (Table S3). In order to determine a % similarity cutoff that can discriminate between the two targets, the two genomes were cut into non-overlapping 150 bp fragments to simulate the most common sequence read length in current metagenome projects, and mapped back to the intact “*Ca.* D. cowenii” and *“Ca.* D. audaxviator” genomes using FR-HIT with default parameters, restricting matches to the single top best hit. Percent similarities ranging from 70–100% were tested in one percent increments in order to quantify the frequency that the fragmented genomes map to their source genome. A 96% similarity level was ultimately used because it restricted spurious matches (i.e., reads mapping from one genome to the other) to a frequency of ∼1% (Fig. S1). The ratio of reads mapping to “*Ca.* D. cowenii” or “*Ca.* D. audaxviator” was calculated and visualized using Circos.

### Sample access and affiliated information

The annotated draft genome of “*Ca.* D. cowenii” modA32 is available via the IMG web portal under Taxon ID number 2615840622 (Gold Analysis Project ID: Ga0071115) and NCBI whole genome shotgun (WGS) project MPOA00000000. The U1362A and U1362B metagenomes are available via the IMG-M web portal under Taxon ID numbers 330002481 and 3300002532, respectively. Gold Analysis Project ID numbers are Ga0004278 (U1362A) and Ga0004277 (U1362B). Sample metadata can be accessed using the BioProject identifier PRJNA269163. The NCBI BioSamples used here are SAMN03166137 (U1362A) and SAMN03166138 (U1362B). Raw sequence data can be accessed using NCBI SRA identifiers SRR3723048 (U1362A) and SRR3732688 (U1362B). A FASTA file containing all EMIRGE-reconstructed SSU rRNA genes from the two borehole fluid metagenomes can be accessed at https://doi.org/10.6084/m9.figshare.4539149.v1.

## Results and Discussion

### Bin identification and refinement

**Figure 1:**
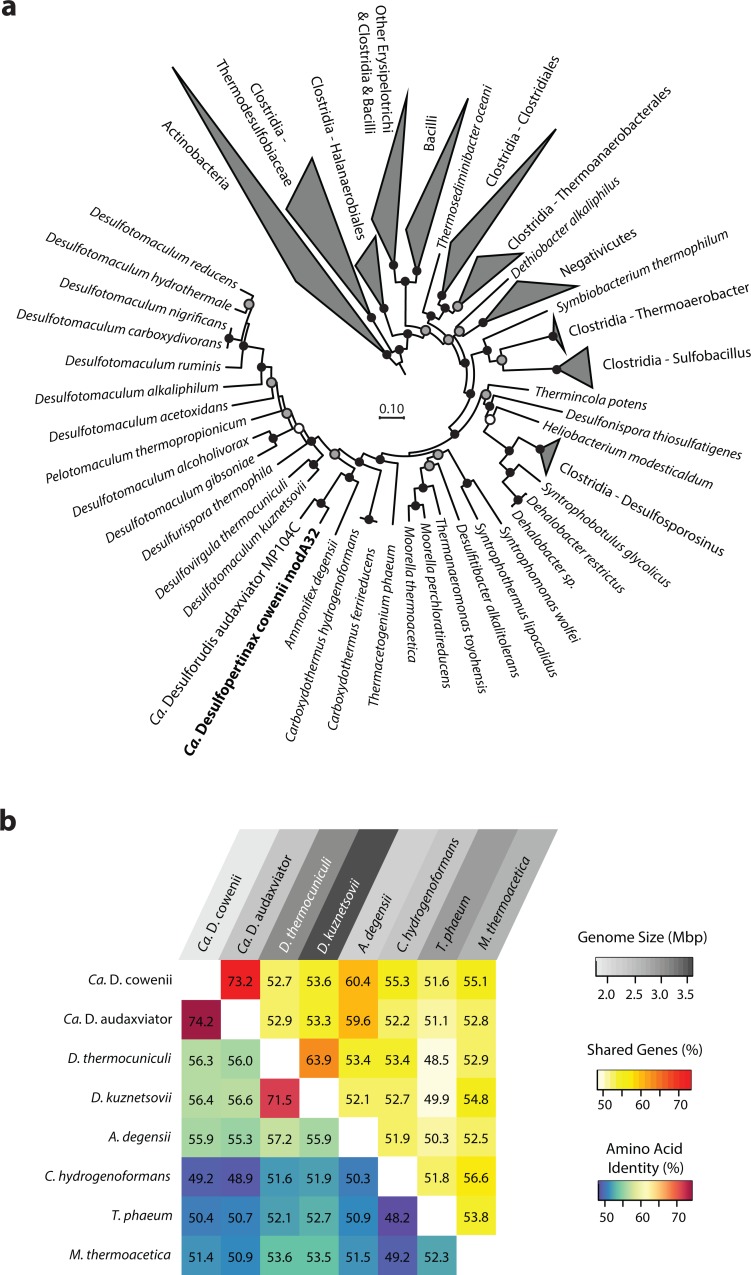
Phylogenomic and shared gene content analysis of “*Ca.* Desulfopertinax cowenii,” “*Ca.* Desulforudis audaxviator” and other Firmicutes. Analysis of phylogenomic relationships, percent shared genes, and average amino-acid identity between “*Ca.* Desulfopertinax cowenii” modA32 and “*Ca.* Desulforudis audaxviator” MP104C reveal two lineages similar to each other and distinct from other *Firmicutes*. (A) Phylogenomic relationships between “*Ca.* D. cowenii,” “*Ca.* D. audaxviator,” and other *Firmicutes* based on a concatenated amino acid alignment of 43 universal single-copy marker genes. Black (100%), gray (>80%), and white (>50%) circles indicate nodes with high local support values, from 1,000 replicates. Actinobacteria (*n* = 687) were used as an outgroup. The scale bar corresponds to 0.10 substitutions per amino acid position. (B) Percent shared genes and average amino-acid identity between “*Ca.* D. cowenii,” “*Ca.* D. audaxviator,” and six closely related *Firmicutes* lineages from (A). The grey scale distinguishing horizontal axis labels corresponds to genome size.

**Figure 2:**
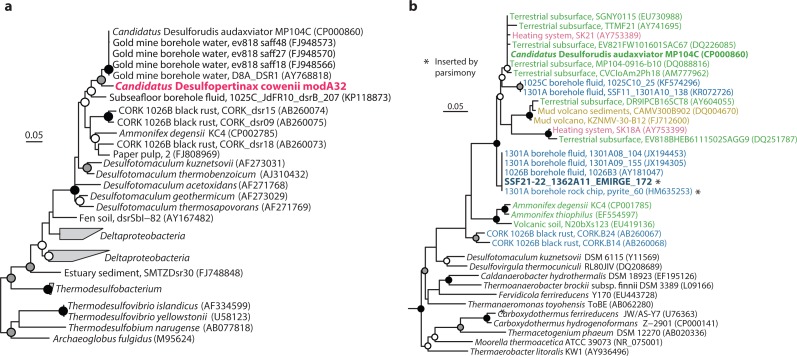
Phylogenetic analysis of “*Ca.* Desulfopertinax cowenii,” “*Ca.* Desulforudis audaxviator” and other closely related *dsrB* and SSU rRNA genes. Phylogenetic relationships between “*Ca.* Desulfopertinax cowenii,” “*Ca.* Desulforudis audaxviator,” and closely related *dsrB* genes (A) and a SSU rRNA gene related to “*Ca.* D. audaxviator” reconstructed from the U1362A metagenome via EMIRGE (B) lend additional support to a shared evolutionary history between “*Ca.* D. cowenii” and “*Ca.* D. audaxviator.” Black (100%), gray (≥80%), and white (≥50%) circles indicate nodes with bootstrap support, from 2,000 replicates. The scale bars correspond to 0.05 substitutions per nucleotide position. SSU rRNA gene sequences are colored according to their source location: blue, marine igneous basement; yellow, marine sediments; green, terrestrial subsurface; red, artificial (man-made).

**Figure 3:**
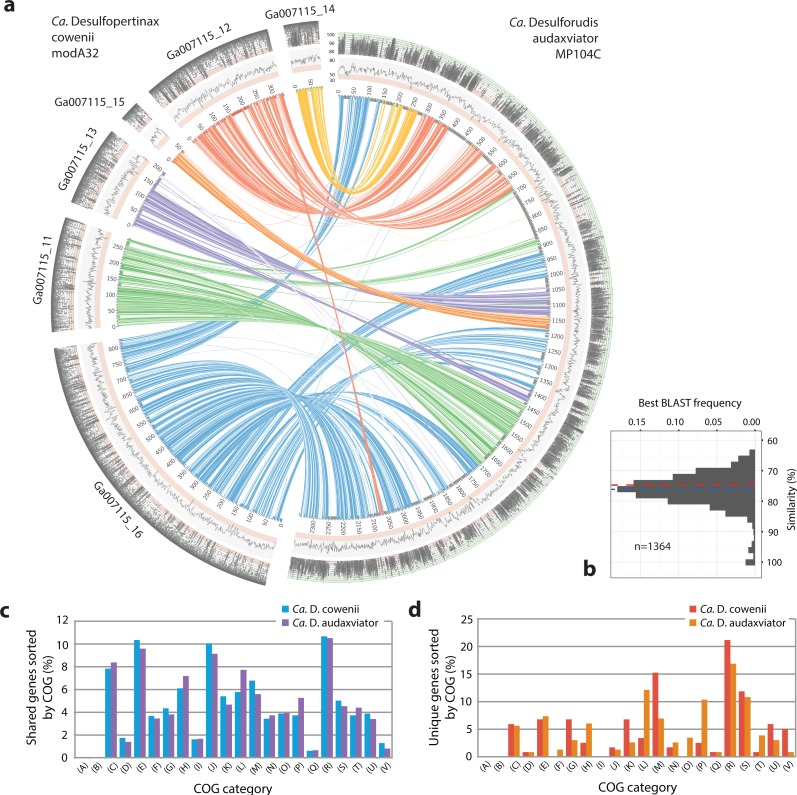
Analysis of genome alignment and shared and unique gene inventories in “*Ca.* Desulfopertinax cowenii” and “*Ca.* Desulforudis audaxviator.” Multiple genome alignment and analysis of shared and unique gene inventories reveal key conserved and variable features of “*Ca.* Desulfopertinax cowenii” and “*Ca.* Desulforudis audaxviator.” (A) Comparison of the “*Ca.* D. cowenii” genome scaffolds with “*Ca.* D. audaxviator” based on reciprocal best BLAST. From innermost to outermost, concentric circles show: nucleotide positions of genomes and scaffolds, percent GC content using a 100 bp sliding window, similarity of mapped U1362A reads. Links connecting circles are colored according to “*Ca.* D. cowenii” scaffold origin [Ga007115_(11–16)] and the degree of shading represents similarities (minimum similarity 70%) based on BLAST comparisons using <75% (light shade), ≥75% (dark shade) nucleic acid identity thresholds. (B) Frequency of reciprocal best BLAST hits (*n* = 1, 364) by percent similarity. Percent similarity histogram bins are in 2% increments and the dashed lines indicate average nucleotide identity (red) and average amino acid identity (blue) between “*Ca.* D. cowenii” and “*Ca.* D. audaxviator.” Relative abundance of shared (C) and unique (D) genes in the “*Ca.* D. cowenii” and “*Ca.* D. audaxviator” genomes, sorted by annotated COG categories. COG categories are: (A) RNA processing and modification; (B) Chromatin structure and dynamics; (C) Energy production and conversion; (D) Cell cycle control, cell division, chromosome partitioning; (E) Amino acid transport and metabolism; (F) Nucleotide transport and metabolism; (G) Carbohydrate transport and metabolism; (H) Coenzyme transport and metabolism; (I) Lipid transport and metabolism; (J) Translation, ribosomal structure and biogenesis; (K) Transcription; (L) Replication, recombination and repair; (M) Cell wall/membrane/envelope biogenesis; (N) Cell motility; (O) Post-translational modification, protein turnover, and chaperones; (P) Inorganic ion transport and metabolism; (Q) Secondary metabolites biosynthesis, transport, and catabolism; (R) General function prediction only; (S) Function unknown; (T) Signal transduction mechanisms; (U) Intracellular trafficking, secretion, and vesicular transport; (V) Defense mechanisms.

Of 60 and 41 genome bins representing diverse groups of uncultivated bacteria and archaea reconstructed from the U1362A and U1362B metagenomes, respectively, one that comprised a nearly complete genome from U1362A (bin A32) was identified as related to “*Ca.* D. audaxviator” by phylogenetic analyses of a set of concatenated single copy marker genes. In order to maximize genome recovery while minimizing potential contamination, contigs within genome bin A32, the “*Ca.* D. audaxviator” genome, and scaffolds related to “*Ca.* D. audaxviator” that were assembled directly from the U1362A and U1362B metagenomes were used as references for mapping raw sequence reads from the U1362A and U1362B metagenomes via several read mapping methods. Because of the relatively high abundance of reads in the U1362A library compared to U1362B, sequence mate pairs from the U1362A metagenome that mapped to these templates were pooled and reassembled (Table S1). Following subsequent screening and removal of contaminating sequences (Table S4), six genomic scaffolds from U1362A totaling 1,778,734 base pairs (bp) in length and originating from only the U1362A metagenome were identified that correspond to the draft “*Ca.* D. cowenii” modA32 genome described here (Table 1). Average read coverage of “*Ca.* D. cowenii” modA32 was 55.0. The purity of the modified genomic bin was supported by results generated using CheckM (Parks et al., 2015) (Table 2), congruent phylogenetic analyses of concatenated marker genes (Fig. 1A) and *dsrB* (Fig. 2A) and *dsrA* genes (Fig. S2), and a high percent of shared genes and gene synteny between the six genomic scaffolds of “*Ca.* D. cowenii” and the “*Ca.* D. audaxviator” genome (Figs. 1B and 3A).

**Table 1:** Genome characteristics of “*Ca.* Desulfopertinax cowenii” modA32 and “*Ca.* Desulforudis audaxviator” MP104C.

	*“Ca.* D. cowenii”	*“Ca.* D. audaxviator”
Percent complete	98–99% (6 scaffolds)	100% (closed)
Genome size (bp)	1,778,734	2,349,476
Percent coding	89.8%	87.6%
GC content	60.2%	60.9%
Total no. of genes	1,842	2,293
No. of protein coding genes	1,782 (96.7%)	2,239 (97.6%)
With function prediction	1,518 (85.2%)	1,587 (70.9%)
Without function prediction	264 (14.8%)	652 (29.1%)
Shared	1,514 (85.0%)	1,606 (71.7%)
Paralogs	137	265
Pseudogenes	n.d.	82
rRNA genes	2	6
5S rRNA	2	2
16S rRNA	n.d.	2
23S rRNA	n.d.	2
tRNA genes	44	45
CRISPR elements	1	4
Mobile elements (integrases/transposons)	6/7	23/81

**Notes.**

TITLE n.d. not detected

**Table 2:** “*Ca.* Desulforudis audaxviator” MP104C-related genome bins from the U1362A metagenome, analyzed by CheckM.

Bin_ID	Total contigs/N50 (Kbp)/ longest contig (Kbp)	Completeness (%)	Contamination (%)	Strain heterogeneity (%)	Total bases (Mbp)
D. audaxviator	–	98.09	0.32	0	2.35
1362A_maxbin32	50/112/179	97.61	5.10	100	1.87
1362A_maxbin32 (ProDeGe filtered)	31/112/179	95.70	5.10	100	1.81
“*Ca.* D. cowenii” modA32 (SPAdes reassembly, ProDeGe filtered)	6/332/826	97.61	0	0	1.78

The 1.78 Mbp “*Ca.* D. cowenii” modA32 genome is 98–99% complete based on separate analyses of tRNA and other marker gene content specific to the phylum *Firmicutes* (Table 1). A phylogenomic analysis of 43 conserved marker genes confirmed a monophyletic relationship between “*Ca.* D. cowenii” and “*Ca.* D. audaxviator” within the *Firmicutes* (Fig. 1A), a relationship that was also supported by analyses of both *dsrA* (Fig. S2) and *dsrB* genes (Fig. 2A). While no small-subunit (SSU) rRNA genes were identified in the “*Ca.* D. cowenii” genome bin, a single full-length SSU rRNA gene related to “*Ca.* D. audaxviator” was reconstructed from raw U1362A metagenome reads. Phylogenetic analyses revealed this gene to form a tight cluster with SSU rRNA genes recovered previously from the deep subseafloor along the Juan de Fuca Ridge flank and, more broadly, a monophyletic lineage with “*Ca.* D. audaxviator” within the phylum *Firmicutes* (Fig. 2B). Consistent with previous studies (Jungbluth et al., 2014; Jungbluth et al., 2016), oceanic crustal fluid SSU rRNA gene clones formed at least two independent sub-lineages within this clade (Fig. 2B). Overall, the topology of the 16S rRNA, *dsrA*, and *dsrB* gene phylogenies reveal multiple distinct lineages related to the Ammonifex, *Ca.* D. audaxviator, *Ca*. D. cowenii, and several additional uncharacterized lineages containing members from the marine and terrestrial deep subsurface. Additional genomic information from these Firmicute lineages will help reveal the functional and evolutionary characteristics shared among deep subsurface microbial life.

### Comparative genomics

The genomes of “*Ca.* D. cowenii” and “*Ca.* D. audaxviator” share an average nucleotide identity of 76.9%. This similarity value is 7% higher than “*Ca.* D. cowenii” shares with the next most closely related Firmicute genomes and demonstrates that “*Ca.* D. cowenii” and “*Ca.* D. audaxviator” originate from, at least, different species (Konstantinidis & Tiedje, 2005a). Similarly, the genomes of “*Ca.* D. cowenii” and “*Ca.* D. audaxviator” share an average amino acid identity of 74.2%, nearly 18% higher than “*Ca.* D. cowenii” shares with its next most similar genome, the Firmicute *Desulfotomaculum kuznetsovii* DSM 6115 (Fig. 1B). In addition to reinforcing the species-level evolutionary divergence observed with ANI, an AAI value of 74.2% indicates that the genomes of “*Ca.* D. cowenii” and “*Ca.* D. audaxviator” lie at the boundary demarcating genus-level divergence (Konstantinidis & Tiedje, 2005b; Konstantinidis & Tiedje, 2007). A similar result was obtained by quantifying the proportion of genes shared between “*Ca.* D. cowenii” and “*Ca.* D. audaxviator” (73.2%) (Fig. 1B).

Compared to the genomes of its closest relatives, the 1.78 Mbp genome harbored by “*Ca.* D. cowenii” is small (Fig. 1B). Despite the smaller size of the “*Ca.* D. cowenii” genome compared to the 2.35 Mbp genome of “*Ca.* D. audaxviator,” the two share similar coding density (89.8% vs. 87.6%), resulting in 451 fewer genes in “*Ca.* D. cowenii” (1,842 vs. 2,293) (Table 1). Compared to other firmicutes, the predicted genome size of “*Ca.* D. cowenii” is among the smallest for members of the Class *Clostridia* with an elevated %GC (Fig. S3); this relatively small genome size might be expected given the low flux of energy and nutrients in the deep subseafloor environment . The smaller genome of “*Ca.* D. cowenii” shares 1,514 of its 1,782 (85.0%) protein coding genes with “*Ca.* D. audaxviator.” Despite the lower gene content overall, “*Ca.* D. cowenii” harbors a similar number of protein coding genes with a predicted function as the genome of “*Ca.* D. audaxviator” (1518 vs. 1587) (Table 1). In addition to a smaller genome and fewer genes, “*Ca.* D. cowenii” also contained fewer pseudogenes (0 vs. 82) and paralogs (137 vs. 265) in comparison to “*Ca.* D. audaxviator” (Table 1), which together suggest some form of streamlining of the “*Ca.* D. cowenii” genome. Compared to “*Ca.* D. audaxviator,” the genome of “*Ca.* D. cowenii” contains fewer CRISPR elements, integrases and transposases, and phage-related genes, which suggests lower viral infection and less horizontal gene transfer in the marine lineage.

**Figure 4:**
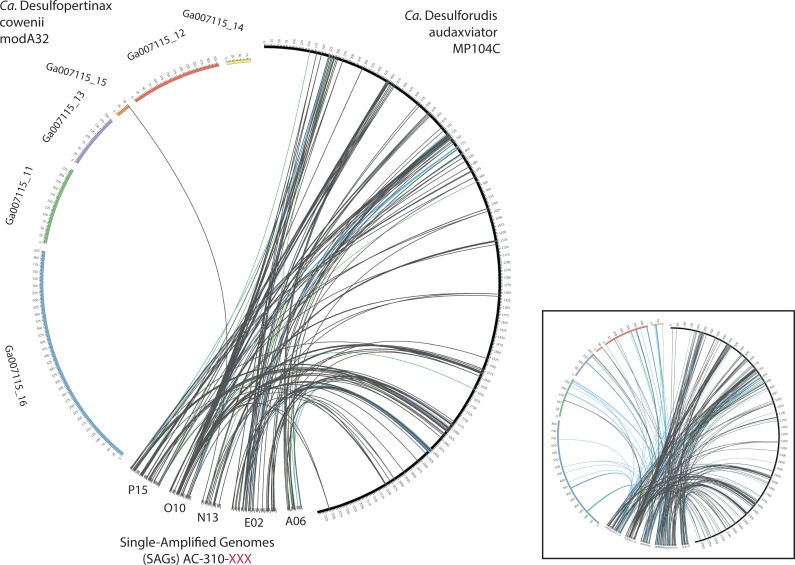
Analysis of genome alignment between “*Ca.* Desulfopertinax cowenii,” “*Ca.* Desulforudis audaxviator” and five closely related single-cell genomes. Comparison of terrestrial deep subsurface SAGs AC-310-P15, O10, N13, E02, and A06 with the genomes of “*Ca.* Desulfopertinax cowenii” and “*Ca.* Desulforudis audaxviator.” Links connecting colored circles represent similarities based on blastn comparisons allowing a maximum of one best hit and using 75–80% (green), 80–85% (blue), >85% (grey) nucleic acid identity thresholds. Inset plot indicates blastn comparisons allowing a maximum of a two best hits.

Extensive gene synteny between “*Ca.* D. cowenii” and “*Ca.* D. audaxviator” was revealed by comparing locations of homologs (Figs. 3A and 3B). Aligning the genome of “*Ca.* D. cowenii” with five incomplete (3.6–7.8% complete) single amplified genomes (SAGs) isolated from the terrestrial South Africa subsurface and related to “*Ca.* D. audaxviator” (Labonté et al., 2015) revealed that all of the SAGs were more similar to “*Ca.* D. audaxviator” than “*Ca.* D. cowenii” (Fig. 4).

### Similarities in functional gene complement

Comparisons of predicted proteins assigned to clusters of orthologous groups (COGs) revealed a markedly similar distribution within the “*Ca.* D. cowenii” and “*Ca.* D. audaxviator” genomes (Fig. 3C). A detailed description of these shared features is included in Table S5.

The genome of “*Ca.* D. cowenii” reveals a microorganism that is functionally similar to “*Ca.* D. audaxviator”: an independent lifestyle consisting of a motile, sporulating, thermophilic, anaerobic chemolithoautroptroph genetically capable of dissimilatory sulfate reduction, hydrogenotrophy, carbon fixation via the reductive acetyl-coenzyme A (Wood-Ljungdahl) pathway, and synthesis of all amino acids. The genome of “*Ca.* D. cowenii” also indicates a chemoorganotroph that possesses abundant sugar transporters and is capable of glycolysis, which is somewhat surprising given the low dissolved organic carbon concentrations in this system (Lin et al., 2012). Similar to “*Ca.* D audaxviator,” hydrogenases were abundant in “*Ca.* D. cowenii,” which is consistent with the availability of hydrogen in basement fluids of the Juan de Fuca Ridge flank (Lin et al., 2014). Altogether, the shared features between “*Ca.* D. cowenii” and “*Ca.* D. audaxviator” help to explain the wide distribution of this lineage in the global deep subsurface.

### Differences in functional gene complement

Despite highly similar genomes overall, comparisons of predicted proteins assigned to clusters of orthologous groups (COGs) revealed unique genes in “*Ca.* D cowenii” that were not found in “*Ca.* D. audaxviator” (Fig. 3D; also see Tables S6 and S7). These genes are likely locations to uncover features that differentiate the marine versus terrestrial members of this lineage. While most unique genes in the “*Ca.* D. cowenii” genome have general functional characterizations only (COG category R), the largest fraction of unique genes in the “*Ca.* D. cowenii” versus “*Ca.* D. audaxviator” genome are found within COG category M (Cell wall/membrane/envelop biogenesis) and include nucleoside-diphosphate-sugar epimerases (e.g., *galE*) and glycosyltransferases (e.g., *treT*) involved in cell wall biosynthesis, and possibly in the production of exopolysaccharides involved with biofilm formation. Defense mechanisms (COG category V) contained the highest ratio of unique genes in the “*Ca.* D. cowenii” genome compared to “Ca D. audaxviator” and includes genes related to ABC-type multidrug transport systems, multidrug resistance efflux pumps (*hylD*), and a class-A beta-lactamase. The marine genome has numerous monosaccharide transporters not present in the terrestrial genome, including those encoding for components of ribose/xylose, arabinose, methyl-galactoside, xylose, allose, and rhamnose transport. Thus, potential differences in organic carbon substrate specificity are evident, which might be expected given the different ages and reactivity of organic material in the marine and terrestrial deep subsurface (e.g., Lang et al., 2006; Simkus et al., 2016).

**Figure 5:**
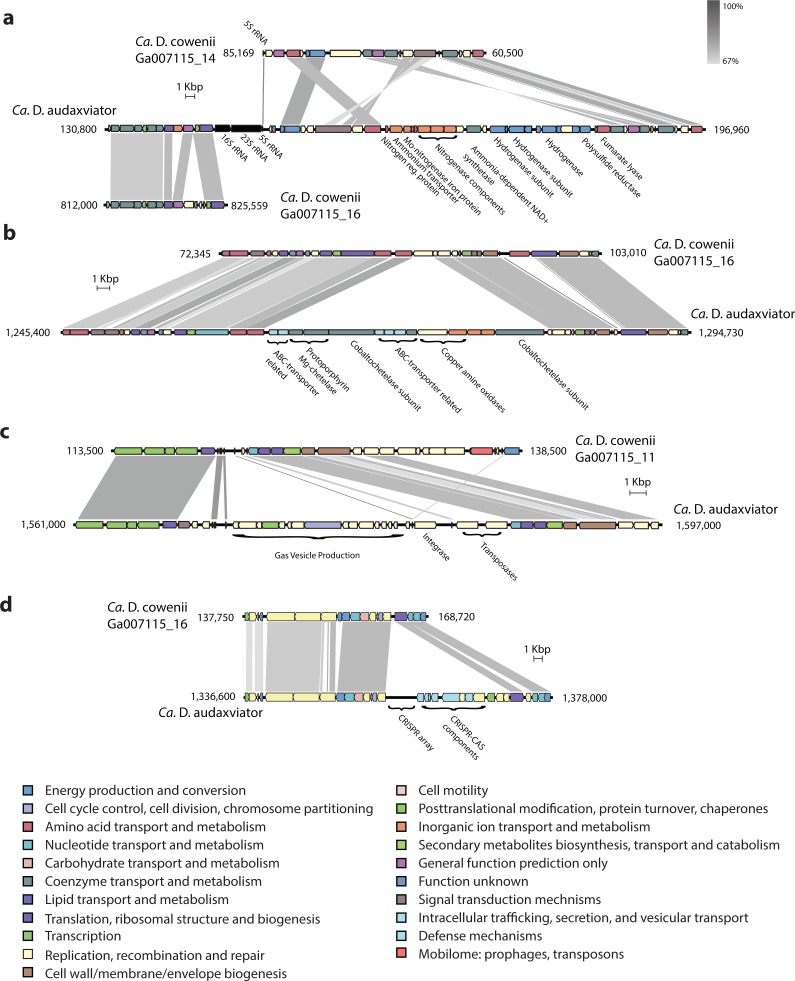
Comparative analysis of genomic organization in “*Ca.* Desulfopertinax cowenii” and “*Ca.* Desulforudis audaxviator.” Comparison of genomic organization in “*Ca.* Desulfopertinax cowenii” with “*Ca.* Desulforudis audaxviator” highlighting regions with large, internal insertion/deletion events containing no homologous genes in the opposing genome. (A) nitrogen-fixation operon, (B) vitamin B12 synthesis, (C) gas vesicle production, (D) a CRISPR-CAS array. Genes are colored according to COG categories and BLAST similarity between regions is indicated by shading intensity.

Though the genome of “*Ca.* D. cowenii” is incomplete, within assembled contigs there are a small number of large indels that are also potential sources of functional differentiation between “*Ca*. D cowenii” and “*Ca.* D. audaxviator.” An indel present in “*Ca.* D. audaxviator” but lacking in “*Ca.* D. cowenii” includes a nitrogenase operon as well as genes for ammonium transport and nitrogen regulation (Fig. 5). While the genes for glutamine synthetase and glutamate synthase within the genome of “*Ca.* D. cowenii” suggest that it obtains its nitrogen from the abundant ammonia in Juan de Fuca Ridge flank crustal fluids (Lin et al., 2012), it appears to be unable to fix inorganic dinitrogen. Another indel suggests that “*Ca.* D. cowenii” lacks the capacity to produce cobalamin (Fig. 5). Moreover, a large cassette of genes present in the “*Ca.* D. audaxviator” genome that is related to gas vesicle production (and flanked by an integrase and two transposases) is missing in “*Ca.* D. cowenii.” Finally, CRISPR-CAS gene arrays and CRISPR elements were distinct between the two genomes (Fig. 5), with the genome of “*Ca.* D. cowenii” encoding 14 CRISPR-associated proteins versus 25 in “*Ca.* D. audaxviator.”

### Distribution

**Figure 6:**
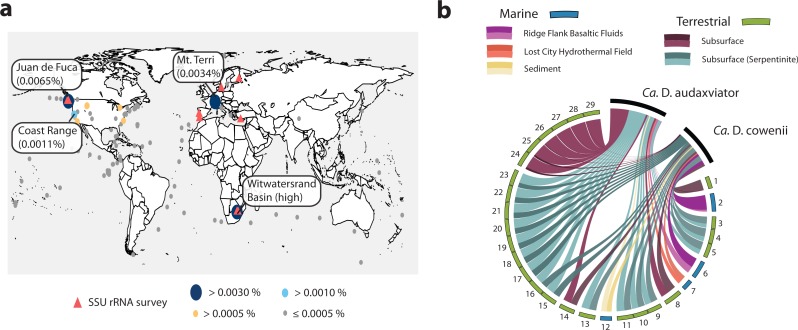
Analysis of the global distribution of “*Ca.* Desulfopertinax cowenii” and “*Ca.* Desulforudis audaxviator.” “*Ca.* Desulfopertinax cowenii” and ”*Ca.* Desulforudis audaxviator” are globally-distributed in the deep subsurface. (A) Ellipse sizes correspond to the frequency of mapped reads from environmental metagenomes to “*Ca.* D. cowenii” and “*Ca.* D. audaxviator” genomes. Triangles indicate locations where a lineage has been detected in SSU rRNA gene surveys. The average frequency of reads mapped to “*Ca.* D. coweii” and “*Ca.* D. audaxviator” are shown for all metagenomes listed in Table S2 with >50,000 genes. (B) Graphical representation of the frequency of environmental genome reads mapping to the “*Ca.* D. cowenii” and “*Ca.* D. audaxviator” genomes using a 96% read similarity score. Environmental metagenomes with the highest ratio of reads mapped to “*Ca.* D. cowenii” vs. “*Ca.* D. audaxviator” and having an average frequency of ≥0.00025 mapped reads are ordered in clockwise fashion from highest to lowest (Table S2). MG-RAST metagenome 4440282 was retained solely because it had the highest ratio of reads mapped to “*Ca.* D. cowenii”:“*Ca.* D. audaxviator.” Links are colored according to the environmental source of each metagenome, while link sizes are proportional to the frequency of a read from a metagenome to map to one genome or the other. The log of metagenome size (number of reads) was used to create the relative length of the outer edges of the circle, which coarsely divide the environments into marine versus terrestrial. The “*Ca.* D. cowenii” genome is sized 2.2× the largest displayed metagenome and “*Ca.* D. audaxviator” is 1.32× (ratio of genome sizes) larger than the “*Ca.* D. cowenii” genome.

The Desulfopertinax/Desulforudis lineage was detected in metagenomic data generated from the terrestrial subsurface of Mt. Terri, Switzerland and the Coast Range Ophiolite, California, USA (Fig. 6A; see also Table S2). It was also found within marine sediments from the coastal Atlantic and Pacific, a Yellowstone National Park hot spring, and the terrestrial subsurface in Ontario, Canada, but never identified in seawater worldwide. Mapping raw metagenome reads in a lineage-specific manner that discriminated between reads mapping to “*Ca.* D. audaxviator” and “*Ca.* D. cowenii” revealed partitioning of these genomes between terrestrial and marine environments, respectively (Fig. 6B; see also Table S3). Surprisingly, the ratio of mapped reads from “*Ca.* D. cowenii” to “*Ca.* D. audaxviator” was, highest (18.9) in a sample from the terrestrial subsurface. The next largest ratios were from the U1362A metagenome (7.3), three serpentinite groundwater metagenomes (1.7–1.6), and the U1362B metagenome (1.4). The ratio of “*Ca.* D. audaxviator” to “*Ca.* D. cowenii” reads was highest (up to ∼165) in samples collected from the terrestrial subsurface of Witwatersrand Basin, South Africa, although this lineage also appears present in serpentinite fluids from the terrestrial subsurface. Thus, it appears that the Desulfopertinax/Desulforudis lineage has a cosmopolitan distribution throughout the global subsurface environment, as indicated by mapping reads from 489 metagenomes from the terrestrial and marine subsurface to the genomes of “*Ca.* D. cowenii” and “*Ca.* D. audaxviator,” as well as gene clones identified in published SSU rRNA surveys (Fig. 6; see also Fig. 2B and Tables S2 and S3).

## Conclusions

Crustal fluids within the terrestrial and marine deep subsurface contain microbial life living at the biosphere’s limit; globally, deep subsurface biosphere is thought be one of the largest reservoirs for microbial life on our planet. This study takes advantage of new sampling technologies and couples them with improvements to DNA sequencing and associated informatics tools in order to reconstruct the genome of an uncultivated *Firmicutes* bacterium from fluids collected deep within the subseafloor of the Juan de Fuca Ridge flank that has previously been documented within both the terrestrial and marine subsurface. Based on our analyses, the capacity for both autotrophic and heterotrophic lifestyles combined with motility and sporulation confers upon “*Ca.* D. cowenii” and “*Ca.* D. audaxviator” the ability to colonize the global deep biosphere. The close shared ancestry between the marine “*Ca.* D. cowenii” and terrestrial “*Ca.* D. audaxviator” provide a unique opportunity to advance our understanding of subsurface microbiology. By comparing the genome of this microorganism to a terrestrial counterpart, we reveal a high and unsuspected degree of functional similarity spanning the marine and terrestrial members of this lineage. Based on the predicted ability to reduce sulfate for energy generation, the persistent detection of this lineage in deep marine biosphere studies, and its initial discovery by deep subseafloor pioneer James Cowen (Cowen et al., 2003), we propose the name “Desulfopertinax cowenii” for this candidatus taxon.

##  Supplemental Information

10.7717/peerj.3134/supp-1 Figure S1 Analysis of mapping frequency of artificially-fragmented reads corresponding to the genomes the “*Ca.* Desulfopertinax cowenii” and “*Ca.* Desulforudis audaxviator” Comparison of mapping frequency of artificially-fragmented 150 bp reads corresponding to the genomes the “*Ca.* Desulfopertinax cowenii” and “*Ca.* Desulforudis audaxviator” mapped to the opposite genome using a range of nucleotide similarity scores. Inset plot shows details between mapping similarity score of 95–100% and revealed a mapping similarity score of 96% restricted spurious matches to a frequency of 1%.

10.7717/peerj.3134/supp-2 Figure S2 Phylogenetic analysis of “*Ca.* Desulfopertinax cowenii,” “*Ca.* Desulforudis audaxviator” and other closely related *dsrA* genes Phylogenetic relationships between “*Ca.* Desulfopertinax cowenii,” “*Ca.* Desulforudis audaxviator,” and closely related *dsrA* genes. Black (100%), gray (≥80%), and white (≥50%) circles indicate nodes with bootstrap support, from 2,000 replicates. The scale bar corresponds to 0.05 substitutions per nucleotide position.

10.7717/peerj.3134/supp-3 Figure S3 Survey of *Firmicutes* genome characteristics Survey of *Firmicutes* genome size, genome GC content, and coding density separated by different classes (*Bacilli*, *Clostridia*, *Erysipelotrichi*, *Negativicutes*). Only complete genomes and genomes with GC content >20% were used (*n* = 909). The genome size of “*Ca.* Desulfopertinax cowenii” was estimated by assuming the current genome length (1.78 Mbp) was 98% the total genome length. Classes are distinguished by shape, while genome size is indicated by shape size and color. All genomes were downloaded from IMG on December 13, 2015.

10.7717/peerj.3134/supp-4 Table S1 Summary of metagenome sequence reads mapped to “*Ca.* Desulforudis audaxviator,” “*Ca.* D. audaxviator”-related scaffolds from IMG-M, and genomic bin A32 from metagenome U1362A

10.7717/peerj.3134/supp-5 Table S2 Metagenomes from the IMG database used in Fig. 6A

10.7717/peerj.3134/supp-6 Table S3 Metagenomes with accessible raw reads from IMG, MG-RAST, and NCBI databases used in Fig. 6B

10.7717/peerj.3134/supp-7 Table S4 Genomic bin purification using ProDeGe

10.7717/peerj.3134/supp-8 Table S5 Similarities in “*Ca.* Desulforudis audaxviator” and “*Ca.* D. audaxviator” by COG category with example genes

10.7717/peerj.3134/supp-9 Table S6 Genes in “*Ca.* D. cowenii” without homologs in “*Ca.* D. audaxviator” genome

10.7717/peerj.3134/supp-10 Table S7 Genes in “*Ca.* D. audaxviator” without homologs in “*Ca.* D. cowenii” genome

10.7717/peerj.3134/supp-11 Supplemental Information 1 “*Ca.* Desulfopertinax cowenii” genome in GenBank format

## Additional Information and Declarations

### DNA Deposition

The following information was supplied regarding the deposition of DNA sequences: The genome sequences described here are accessible via IMG Genome ID 2615840622.

### Data Availability

The following information was supplied regarding data availability: The raw sequence data used for this analysis are accessible via SRA accession numbers SRR3723048 (U1362A) and SRR3732688 (U1362B).
