# Supplementary figures and images for: Genomic comparisons of a bacterial lineage that inhabits both marine and terrestrial deep subsurface systems
Source: PeerJ. 2017 Apr 6;5:e3134. doi: 10.7717/peerj.3134 (PMC5385130; doi:10.7717/peerj.3134)

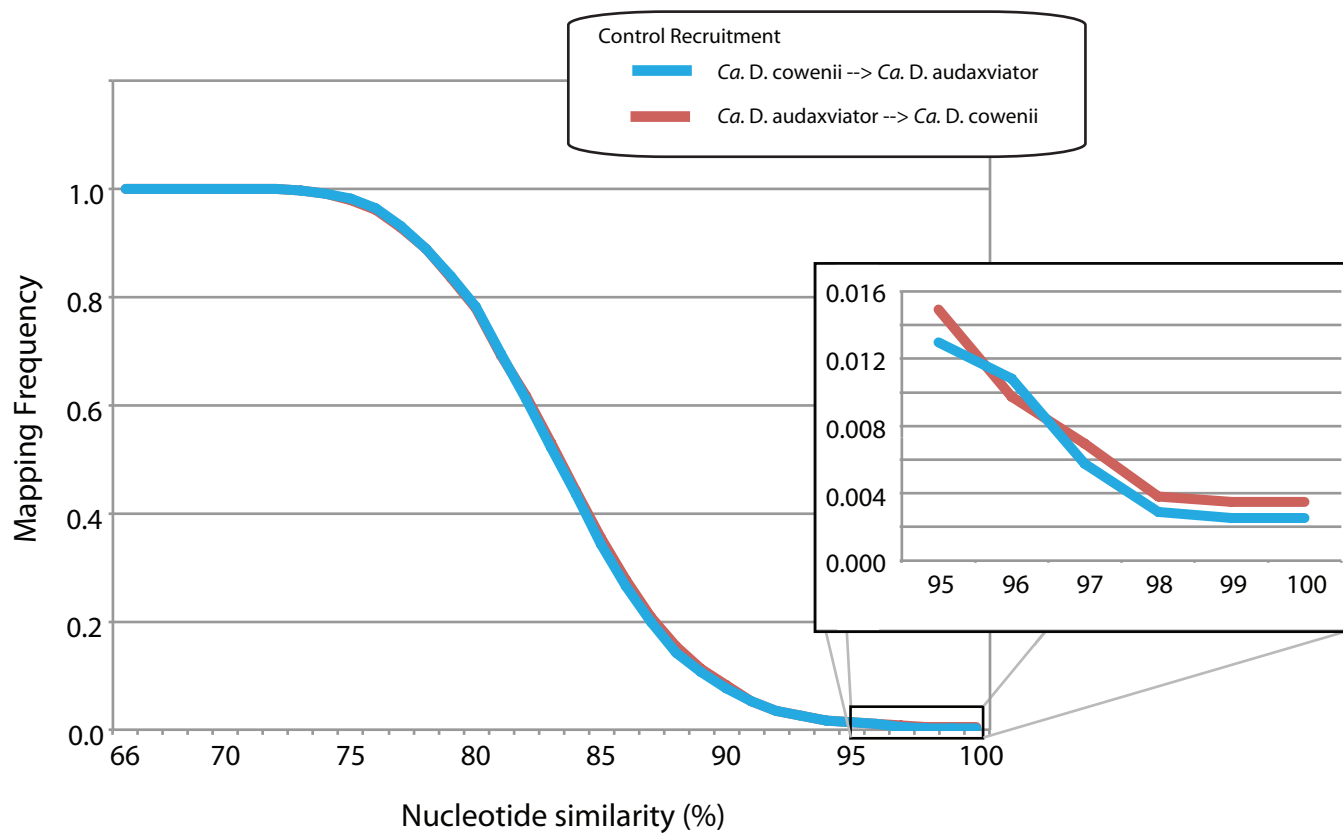

Supplement: Figure S1 — Comparison of mapping frequency of artificially-fragmented 150 bp reads corresponding to the genomes the “Ca. Desulfopertinax cowenii” and “Ca. Desulforudis audaxviator” mapped to the opposite genome using a range of nucleotide similarity scores. Inset plot shows details between mapping similarity score of 95–100% and revealed a mapping similarity score of 96% restricted spurious matches to a frequency of 1%. [file peerj-05-3134-s001.pdf]

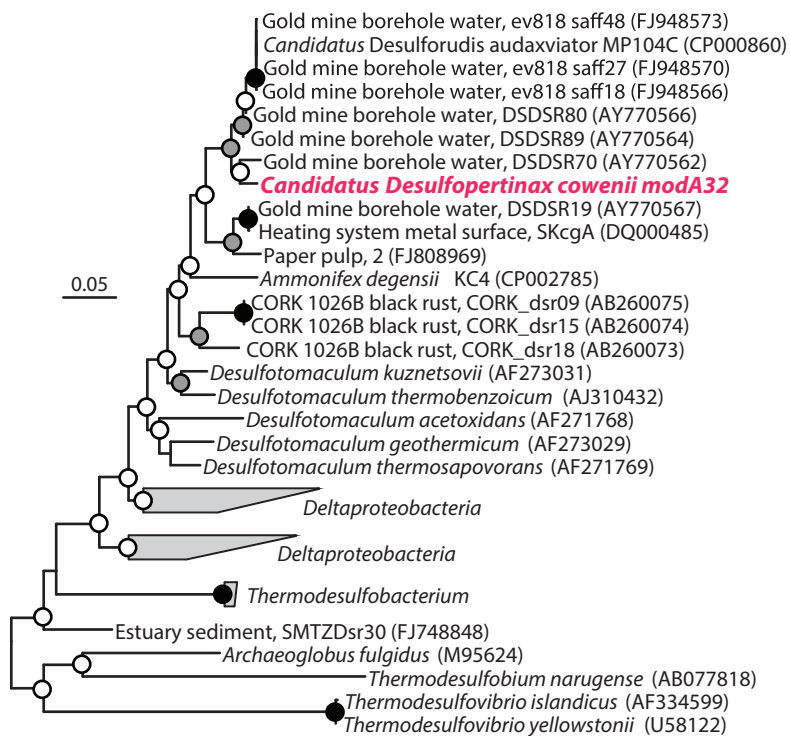

Supplement: Figure S2 — Phylogenetic relationships between “Ca. Desulfopertinax cowenii,” “Ca. Desulforudis audaxviator,” and closely related dsrA genes. Black (100%), gray (≥80%), and white (≥50%) circles indicate nodes with bootstrap support, from 2,000 replicates. The scale bar corresponds to 0.05 substitutions per nucleotide position. [file peerj-05-3134-s002.pdf]

**Class**  
**Firmicutes**

*Bacilli*  
*Clostridia*  
*Erysipelotrichi*  
*Negativicutes*

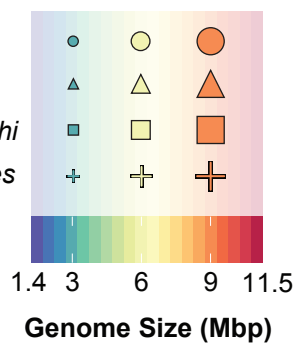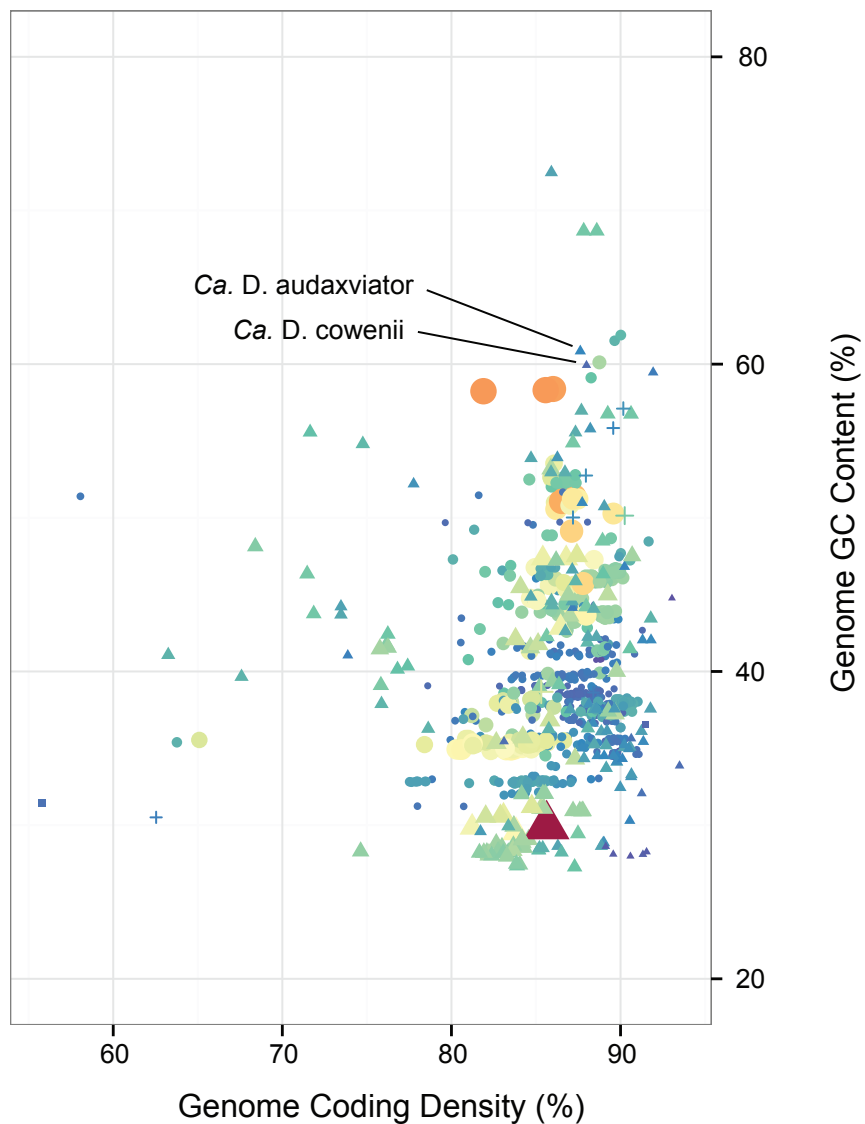

Supplement: Figure S3 — Survey of Firmicutes genome size, genome GC content, and coding density separated by different classes (Bacilli, Clostridia, Erysipelotrichi, Negativicutes). Only complete genomes and genomes with GC content >20% were used (n = 909). The genome size of “Ca. Desulfopertinax cowenii” was estimated by assuming the current genome length (1.78 Mbp) was 98% the total genome length. Classes are distinguished by shape, while genome size is indicated by shape size and color. All genomes were downloaded from IMG on December 13, 2015. [file peerj-05-3134-s003.pdf]
