# Supplementary material for: Genomic comparisons of a bacterial lineage that inhabits both marine and terrestrial deep subsurface systems
Source: PeerJ. 2017 Apr 6;5:e3134. doi: 10.7717/peerj.3134 (PMC5385130; doi:10.7717/peerj.3134)
Supplement: Table S1 [file peerj-05-3134-s004.docx]

Supplementary Table 1. Summary of metagenome sequence reads mapped to “*Ca.* Desulforudis audaxviator”, “*Ca.* D. audaxviator”-related scaffolds from IMG-M, and genomic bin A32 from metagenome U1362A.

| Metagenome | Recruitment method | Target for fragment recruitment | | | Subtotal^b^ | Total^c^ |
| --- | --- | --- | --- | --- | --- | --- |
|  |  | “*Ca.* D. audaxviator” | “*Ca.* D. audaxviator”-related scaffolds | 1362A_maxbin32 |  |  |
| U1362A | bbmap | 524916 (696450)^a^ | 1102222 (1240214) | 778190 (877474) | 1782646 | 1785284 |
|  | Bowtie2 | 40273 (61946) | 857214 (896816) | 585010 (594926) | 1010798 |  |
| U1362B | bbmap | 262753 (365430) | 192421 (270268) | 140984 (203622) | 575918 | 576460 |
|  | Bowtie2 | 22140 (36418) | 63280 (76286) | 29640 (35486) | 117954 |  |

^a^Numbers in parentheses equal the number of reads identified when mate pairs of solo mapped reads are included

^b^Total number of unique reads, including all mate pairs, found via mapping with three references combined

^c^Total number of unique reads, including all mate pairs, found via mapping with three references combined and using the two mapping methods.
