# Supplementary material for: Genomic comparisons of a bacterial lineage that inhabits both marine and terrestrial deep subsurface systems
Source: PeerJ. 2017 Apr 6;5:e3134. doi: 10.7717/peerj.3134 (PMC5385130; doi:10.7717/peerj.3134)
Supplement: Table S4 [file peerj-05-3134-s007.docx]

Supplementary Table 4. Genomic bin purification using ProDeGe.

| Bin_ID | Total contigs | Contam. contigs | Contam. bases (bp) | Clean contigs | Clean bases (Percent of “Ca. D. audaxviator”) |
| --- | --- | --- | --- | --- | --- |
| 1362A_maxbin32 | 50 | 19 | 58450 | 31 (62%) | 1809569 (77%) |
| U1362A recruited reads reassembled with SPAdes | 4276 | 4270 | 5323350 | 6 (<1%) | 1778734 (76%) |
