# Supplementary material for: Genomic comparisons of a bacterial lineage that inhabits both marine and terrestrial deep subsurface systems
Source: PeerJ. 2017 Apr 6;5:e3134. doi: 10.7717/peerj.3134 (PMC5385130; doi:10.7717/peerj.3134)
Supplement: Table S5 [file peerj-05-3134-s008.docx]

Supplementary Table 5. Similarities in “*Ca.* Desulforudis audaxviator” and “*Ca.* D. audaxviator” by COG category with example genes.

|  | COG Categories | Shared Genes |
| --- | --- | --- |
| Glycolysis | G | glucose-6-phosphate isomerase (*pgi*), 6-phosphofructokinase (*pfaA*), fructose-bisphosphate aldolase (*fbaA*), triosephosphate isomerase (*tpiA*), glyceraldehyde-3-phosphate dehydrogenase (*gapA*), phosphoglycerate kinase (*pgk*), enolase (*eno*), pyruvate kinase (*pyk*), phosphoglyceromutase (*gpmI*), fructose-2,6-bisphosphatase (*gpmB*), 6-phosphofructokinase (*pfkA*), glycogen debranching enzyme (*gde*), predicted phosphoglycerate mutase (COG3635), transcriptional regulator/sugar kinase (*nagC*) |
| Gluconeogenesis | G | archaeal fructose 1,6-bisphosphatase (*fbp*), phosphoenolpyruvate synthase/pyruvate phosphate dikinase (*ppsA*), phosphomannomutase (*manB*) |
| Anaerobic electron transport chain and dissimilatory sulfate reduction | C | dissimilatory sulfate reduction encoded by heterodisulfide reductase (*hdrABC*), coenzyme F420-reducing hydrogenase (*qmoABC*), adenylylsulfate reductase (*aprAB*), dissimilatory sulfite reductase (*dsrAB*) |
| Assimilation and fixation of inorganic carbon | C,E,F | formyltetrahydrofolate synthetase (*fhs*), methenyltetrahydrofolate cyclohydrolase (*folD*), 5,10-methylenetetrahydrofolate reductase (*metF*), CODH/acetyl-CoA synthase (*cdhCDE*/*acsABCDE*) |
| Transport functions | E,G,P,R | phosphate (*pstABCS*), sodium (*yfkE*), potassium (*trkAG*), ferrous iron (*feoAB*), cobalt (*cbiMOQ*), magnesium/nickel (*mgtAE*), zinc/manganese (*znuBC*), branched-chain amino acids (*livFGHKM*), polar amino acids (*glnQ*), other amino acids (*hisJM*), dipeptides (*dpdA*/*ddpA*), sugars (*rbsB*), biotin (*bioY*) |
| Amino acid synthesis | E, M | alanine (*alr*), arginine (*argBCDFGHJ*), asparagine (*asnB*), cysteine (*cysEK*), glutamate (*gltB*), glutamine (*glnAD*), glycine (*glyA*), histidine (*hisABCDFGI*), lysine (*lysA*), phenylalanine/tyrosine/tryptophan (*aroABCEHKQ*, *pheA*, *trpABCDEF*, *tyrA*), proline (*proABC*), serine (*serAB*), threonine (*thrAC*), valine/leucine/isoleucine (*leuABCD*, *ilvABCDEH*) |
| Motility (flagella and chemotaxis) | N | *fliCDEFGHIJMNOPQRS*, *flgBCDEGKLMN*, *flhAB*, *motAB*, and *cheABCDRWY* |
| Sporulation | -- | spore coat/cortex (*spsFG*, *yabGQ*) and maturation (*spmAB*) |
